# Burden of chemotherapy‐induced myelosuppression among patients with extensive‐stage small cell lung cancer: A retrospective study from community oncology practices

**DOI:** 10.1002/cam4.5738

**Published:** 2023-03-31

**Authors:** Lowell Hart, Augustina Ogbonnaya, Kristen Boykin, Kathryn Deyoung, Ray Bailey, Trevor Heritage, Lorena Lopez‐Gonzalez, Huan Huang, Lucio Gordan

**Affiliations:** ^1^ Florida Cancer Specialists & Research Institute Florida Fort Myers USA; ^2^ Wake Forest University School of Medicine North Carolina Winston‐Salem USA; ^3^ Xcenda LLC Texas Carrolton USA; ^4^ G1 Therapeutics, Inc. North Carolina Research Triangle Park USA

**Keywords:** chemotherapy, community oncology, extensive‐stage small cell lung cancer, myelosuppression, retrospective, supportive care

## Abstract

**Background:**

Myelosuppression is a major dose‐limiting complication of chemotherapy for patients with extensive‐stage small cell lung cancer (ES‐SCLC). The objective was to describe the burden of myelosuppression, treatment patterns, and supportive care use among patients with ES‐SCLC treated with chemotherapy in a US community oncology setting.

**Methods:**

This retrospective cohort study used structured electronic medical record (EMR) data from the Florida Cancer Specialists & Research Institute between January 2013 and December 2020. Adult patients with ES‐SCLC who were treated with chemotherapy between September 2013 and November 2020 were identified. The index date was the date of the first chemotherapy‐containing line of therapy (LOT). Patients were followed for a minimum of 30 days after index (unless patient died) until December 31, 2020, or end of activity in the EMR data, whichever occurred first. Incidence and frequency of myelosuppressive episodes/events, treatment patterns, eligibility for red blood cell (RBC) or platelet transfusions, and supportive care use (granulocyte colony‐stimulating factor [G‐CSF], erythropoiesis‐stimulating agents [ESAs], intravenous [IV] hydration) during the follow‐up period were reported.

**Results:**

The study population included 1239 patients. Most (94.0%) patients started first‐line chemotherapy at index. During follow‐up and across all chemotherapy‐containing LOTs, 1222 (98.6%) patients had at least 1 myelosuppressive episode; 62.1% of patients had grade ≥ 3 myelosuppressive episodes in at least one lineage, 33.9% had grade ≥ 3 myelosuppressive episodes in at least two lineages, and 15.5% had grade ≥ 3 myelosuppressive episodes in all three lineages. Supportive care use included 89.7% of patients who received G‐CSF, 24.4% who received ESAs, and 52.1% who received IV volume expansion. Almost one‐third (32.6%) of patients were eligible to receive RBC transfusions based on lab values (hemoglobin < 8 g/dL).

**Conclusion:**

There is a high burden related to multilineage myelosuppression among chemotherapy‐treated patients with ES‐SCLC in the community oncology setting. Reducing myelosuppression could make chemotherapy treatment safer, reduce the need for supportive care, and potentially prevent the treatment of complications.

## Graphical abstract

Burden of chemotherapy‐induced myelosuppression among patients with extensive‐stage small cell lung cancer in community oncology practices.

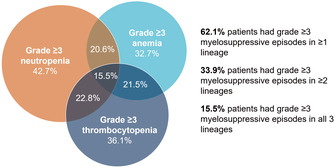

## 1 INTRODUCTION

Small cell lung cancer (SCLC) is an aggressive form of lung cancer characterized by rapid growth and widespread metastasis.^1^, ^2^ SCLC accounts for approximately 15% of all lung cancer cases, and patients often present with extensive‐stage (ES) disease at the time of diagnosis.^1^, ^2^ Most patients with ES‐SCLC in the US are treated in the community setting.^3^ Although SCLC is highly responsive to chemotherapy, early treatment resistance is common, and most patients relapse within the first year after initial therapy.^4^

Standard front‐line treatment for ES‐SCLC comprises of platinum‐based chemotherapy, either alone or in combination with immune checkpoint inhibitors (atezolizumab or durvalumab).^5^, ^6^ Commonly used first‐line chemotherapy combinations include cisplatin/etoposide and carboplatin/etoposide, with a preference for carboplatin over cisplatin owing to the equivalent efficacy of carboplatin and cisplatin and the more tolerable non‐hematologic toxicity profile of the former.^5^, ^7^ For patients who relapse within 6 months, preferred subsequent therapy options include topotecan and lurbinectedin, with other recommended chemotherapy agents including paclitaxel, docetaxel, and irinotecan.^5^ Standard treatments for ES‐SCLC render patients particularly susceptible to hematologic adverse events (AEs).

The side effects associated with systemic chemotherapy regimens, with or without combination immunotherapy, for ES‐SCLC are often severe and dose limiting. Myelosuppression is a frequent complication of cytotoxic chemotherapy that results from damage to hematopoietic stem and progenitor cells in the bone marrow and commonly manifests as neutropenia, anemia, and/or thrombocytopenia.^8^ The degree of damage to white blood cells (WBCs), red blood cells (RBCs), and platelets depends on the specific chemotherapy regimen used and on baseline patient and clinical characteristics.^8^, ^9^, ^10^, ^11^ However, the burden of chemotherapy‐induced myelosuppression (CIM) on patients with cancer is substantial, placing patients at significant risk of serious infections, bleeding, sepsis, and even death.^10^, ^12^, ^13^, ^14^, ^15^ Further, symptoms such as fatigue and concerns over infection risk may have a considerable negative impact on patients' quality of life and may impact their ability to continue treatment.^16^, ^17^

Myelosuppression typically necessitates chemotherapy dose delays, reductions, and discontinuations, potentially resulting in suboptimal treatment outcomes,^18^, ^19^ and the use of supportive care interventions such as growth factors (granulocyte colony‐stimulating factors [G‐CSFs] and erythropoiesis‐stimulating agents [ESAs]), RBC or platelet transfusions, and iron supplementation.^10^, ^20^, ^21^, ^22^ However, these treatments are administered reactively, after the onset of symptoms; even G‐CSF, which is sometimes used prophylactically to boost stem cell growth and neutrophil production, acts after the bone marrow has already sustained damage.^23^ Hematologic AEs related to myelosuppression give rise to higher health care resource utilization (HCRU) and higher health care costs owing to the need for rescue interventions and/or hospitalizations, thus incurring a considerable economic burden on health care systems and patients.^24^, ^25^, ^26^, ^27^, ^28^

There is limited research on burden of CIM among patients with ES‐SCLC. This study described the burden of chemotherapy‐induced myelosuppression, treatment patterns, and supportive care use among patients with ES‐SCLC treated with chemotherapy in a US community oncology setting.

## 2 METHODS

### 2.1 Data source

This was a retrospective cohort study of patients diagnosed with ES‐SCLC receiving treatment in a US‐based community oncology setting. The study used structured EMR data (i.e., predefined data fields) from the Florida Cancer Specialists and Research Institute (FCS) between January 1, 2013, and December 31, 2020. FCS is one of the largest independent medical oncology and hematology practices in the United States including over 80 facilities across the state of Florida and serves nearly 70,000 new patients annually.

The data were deidentified prior to analysis. All data were handled in compliance with the Health Insurance Portability and Accountability Act and the Health Information Technology for Economic and Clinical Health Act of 1996. The Advarra Institutional Review Board (IRB) reviewed and approved the study protocol, and informed consent was not required.

### 2.2 Patient population and study design

**FIGURE 1:**
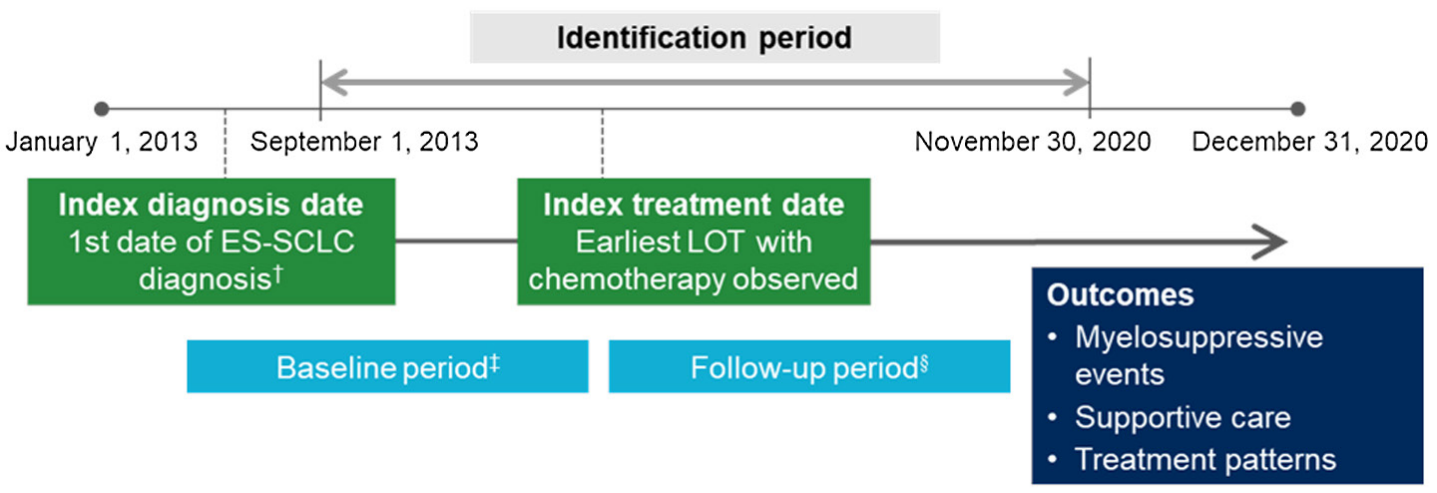
Study diagram. ES‐SCLC, extensive‐stage small cell lung cancer; LOT, line of therapy. ^†^Based on evidence of ES‐SCLC diagnosis in the 180 days prior to the index treatment date for first LOT, or any time prior for second or later LOT. ^‡^Any time prior to the index treatment date. ^§^≥30 days after the index treatment date (patients who died in the first 30 days were included).

The study population included adult (age ≥ 18 years) patients with ES‐SCLC who initiated at least one LOT with chemotherapy between September 1, 2013, and November 30, 2020 (patient identification period; Figure 1). ES‐SCLC diagnosis was defined by all of the following: International Classification of Diseases, 9th Revision, Clinical Modification (ICD‐9‐CM) or 10th Revision (ICD‐10‐CM) diagnosis code for lung cancer (162.x or C34.x, respectively); diagnosis code for metastases (stage IV or ES disease; ICD‐9‐CM = 196.x, 197.x, 198.x, 199.x; ICD‐10‐CM = C77.x, C78.x, or C79.x), and SCLC histology. LOT was defined using the previously validated RefineIQ lining algorithm.

Evidence of ES‐SCLC diagnosis in the 180 days prior to the date of first chemotherapy dose observed during the patient identification period (index treatment date) was required if the index LOT was first‐line therapy. If the index LOT was second‐ or later‐line therapy, ES‐SCLC diagnosis could occur at any time prior to the index treatment date. Patients were followed from before the index treatment date (baseline period) until death, end of activity in the EMR database, or December 31, 2020, whichever occurred first (follow‐up period). Patients were required to have ≥1 encounter in the EMR system after the index treatment date. Exclusion criteria included evidence of other primary cancers (except benign skin cancers) any time during baseline or follow‐up, missing gender or date of birth, <30 days of follow‐up after the index treatment date for reasons other than death, and participation in a clinical trial.

### 2.3 Study measures

Patient demographics and clinical characteristics during the baseline period included age, sex, race, and non‐cancer comorbidities from the Charlson Comorbidity Index.^29^ Eastern Cooperative Oncology Group performance status (ECOG PS) a 5‐point score used to assess functional status and self‐care capabilities^30^ was assessed during the period from 60 days before to 14 days after the index treatment date. Pre‐chemotherapy myelosuppressive events by type and grade were assessed in the 30 days prior to the index treatment date. Myelosuppressive events were identified using ICD‐9‐CM or ICD‐10‐CM diagnosis codes or laboratory values based on Common Terminology Criteria for Adverse Events (CTCAE) version 5.0^31^ definitions for neutropenia (grade 3: absolute neutrophil count [ANC] ≥500 to <1000 cells/μL, grade 4: ANC <500 cells/μL), anemia (grade 3: hemoglobin <8.0 g/dL), thrombocytopenia (grade 3: ≥25,000 to <50,000 platelets/μL, grade 4: <25,000 platelets/μL), lymphopenia (grade 3: ≥1000 to <2000 WBC/μL, grade 4: <1000 WBC/μL), and leukopenia (grade 3: ≥200 to <500 lymphocytes/μL, grade 4: <200 lymphocytes/μL). A myelosuppressive event was defined as each event on a unique date. Grade of myelosuppressive event was defined among patients with laboratory data for the respective myelosuppressive lineage.

The primary measure of interest during follow‐up was the prevalence and frequency of myelosuppressive episodes (by type and grade) across all LOTs. Myelosuppressive episodes were defined as all respective events occurring within 21 days of the first event, with the highest observed grade assigned to that episode. Additional measures during the follow‐up period included time to myelosuppressive episodes (by type), treatment patterns (prevalence of and time to treatment discontinuation and prevalence of and time to next therapy [TTNT]), eligibility to receive RBC (hemoglobin [Hb] <8 g/dL) or platelet (platelets <10,000/μL) transfusion (since transfusion administration was not captured in the data), and supportive care use (G‐CSF administration, ESA administration, and intravenous [IV] hydration) were assessed across all LOTs. Time to myelosuppressive episodes was defined from the start date of the LOT in which the episode occurred. Time to treatment discontinuation (in months) of the index LOT was measured from the index treatment date to the index LOT discontinuation date, the day prior to the start of the subsequent LOT, or death. TTNT was measured as the time from the index treatment date to the date of initiation of a new LOT. Patients who did not discontinue or did not have a new LOT were censored at the end of follow‐up.

### 2.4 Statistical analysis

Descriptive statistics were reported to describe patient characteristics and study measures. Univariate Kaplan–Meier analyses were used to estimate time to myelosuppressive episodes, median time to discontinuation, and median TTNT among all patients. While the main analysis is focused on prevalence and frequency of myelosuppressive episodes across all LOTs, sensitivity analyses were conducted to assess the prevalence of myelosuppressive events during the index LOT and the prevalence and frequency in all LOTs. All analyses were conducted using SAS Enterprise Guide v.8.1 (SAS Institute, Inc., Cary, NC, USA).

## 3 RESULTS

### 3.1 Patient characteristics, pre‐index myelosuppressive events, index LOT

The study population included 1239 patients (Table 1). Mean (standard deviation [SD]) age was 66.9 (9.3) years, 49.7% were male, and 58.0% were White. Most patients (64.5%) had an ECOG PS of 0 (fully active; no performance restrictions) or 1 (strenuous physical activity restricted; fully ambulatory and able to carry out light work). The most common non‐cancer‐related comorbidities from the CCI included chronic pulmonary disease (5.5%), mild liver disease (2.3%), and renal disease (2.2%). Almost all (94.0%) patients started first‐line chemotherapy at the index treatment date. Prior to index chemotherapy initiation, the prevalence of grade ≥ 3 myelosuppressive events was low (neutropenia = 3.5%; anemia = 1.6%; thrombocytopenia = 1.2%; lymphopenia = 2.5%; leukopenia = 2.9%). Mean (SD) follow‐up after index chemotherapy initiation was 10.4 (11.3) months.

**TABLE 1:** Patient demographic and clinical characteristics.

Characteristic^a^	Patients with ES‐SCLC (*N* = 1239)
Age, years, mean (SD) [median]	66.9 (9.3) [68.0]
Age group, *n* (%)	
<65 years	462 (37.3)
≥65 years	777 (62.7)
Male, *n* (%)	616 (49.7)
Race, *n* (%)	
White	719 (58.0)
Black	27 (2.2)
Asian	1 (0.1)
Other	441 (35.6)
Unknown	51 (4.1)
ECOG PS^b^, *n* (%)	
0	299 (24.1)
1	500 (40.4)
2	170 (13.7)
≥3	65 (5.2)
Unknown	205 (16.5)
Continuous non‐cancer‐related CCI, mean (SD)	0.2 (0.5)
Categorical non‐cancer‐related CCI, *n* (%)	
0	1116 (90.1)
1	77 (6.2)
≥2	46 (3.7)
Non‐cancer‐related CCI comorbidities^c^, *n* (%)	
Chronic pulmonary disease	68 (5.5)
Mild liver disease	28 (2.3)
Renal disease	2.2 (2.2)
Myelosuppressive events before index^d^, *n* (%)	
Grade ≥ 3 neutropenia	17 (3.5)
Grade ≥ 3 anemia	8 (1.6)
Grade ≥ 3 thrombocytopenia	6 (1.2)
Grade ≥ 3 lymphopenia	12 (2.5)
Grade ≥ 3 leukopenia	14 (2.9)
Index LOT, *n* (%)	
1	1165 (94.0)
2	71 (5.7)
≥3	3 (0.2)
Follow‐up from index date, days, mean (SD) [median]	10.4 (11.3) [7.3]

Abbreviations: CCI, Charlson Comorbidity Index; ECOG PS, Eastern Cooperative Oncology Group performance status; IO, immuno‐oncology treatment; LOT, line of therapy; SD, standard deviation.

^a^ Measured any time prior to the index treatment date with the exception of ECOG, myelosuppressive events, and follow‐up time.

^b^ Measured within 60 days before or 14 days after the index treatment date. Levels defined as 0 = Fully active, able to carry on all pre‐disease performance without restriction; 1 = Restricted in physically strenuous activity but ambulatory and able to carry out work of a light or sedentary nature; 2 = Ambulatory and capable of all self‐care but unable to carry out any work activities; up and about more than 50% of waking hours; 3 = Capable of only limited self‐care, confined to bed or chair more than 50% of waking hours; 4 = Completely disabled, cannot carry on any self‐care, totally confined to bed or chair; 5 = Dead.

^c^ Limited to comorbidities with prevalence >1%.

^d^ Measured 30 days before the index treatment date.

### 3.2 Myelosuppressive episodes/events

**FIGURE 2:**
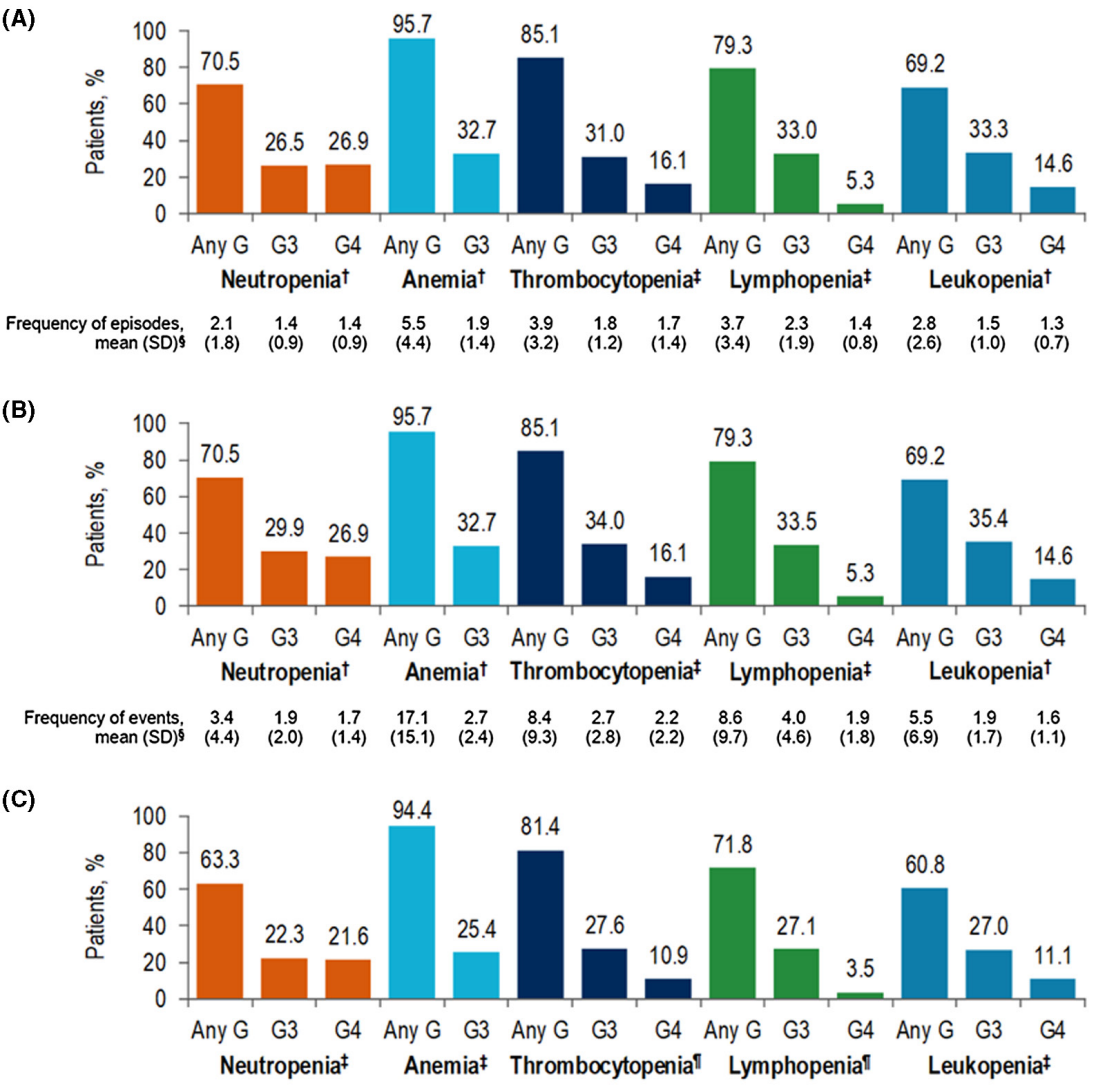
Prevalence and frequency of (A) myelosuppressive episodes across all LOTs (B) myelosuppressive events across all LOTs, and (C) myelosuppressive events during the index LOT. G, grade; LOT, line of therapy; SD, standard deviation. ^†^ *N* = 1236 patients with available laboratory data. ^‡^ *N* = 1235 patients with available laboratory data. ^§^Among patients with ≥1 event across all LOTs. ^¶^ *N* = 1234 patients with available laboratory data.

During follow‐up, 1222 (98.6%) patients had any myelosuppressive episode and about half (48.6%) of patients experienced decreases across all peripheral blood lineages (ie, pancytopenia) across all LOTs. Of the patients with available laboratory data to allow for the grading of CIM events, 26.5% and 26.9% of patients had grade 3 and grade 4 neutropenia, respectively (grade 3 and 4 mean episodes = 1.4), 32.7% had grade 3 anemia (mean episodes = 1.9), and 31.0% and 16.1% had grade 3 and grade 4 thrombocytopenia (grade 3 mean episodes = 1.8; grade 4 mean episodes = 1.7), respectively (Figure 2A). Consistent results were observed when comparing the prevalence of myelosuppressive events during the index LOT and all LOTs (Figure 2B,C).

**FIGURE 3:**
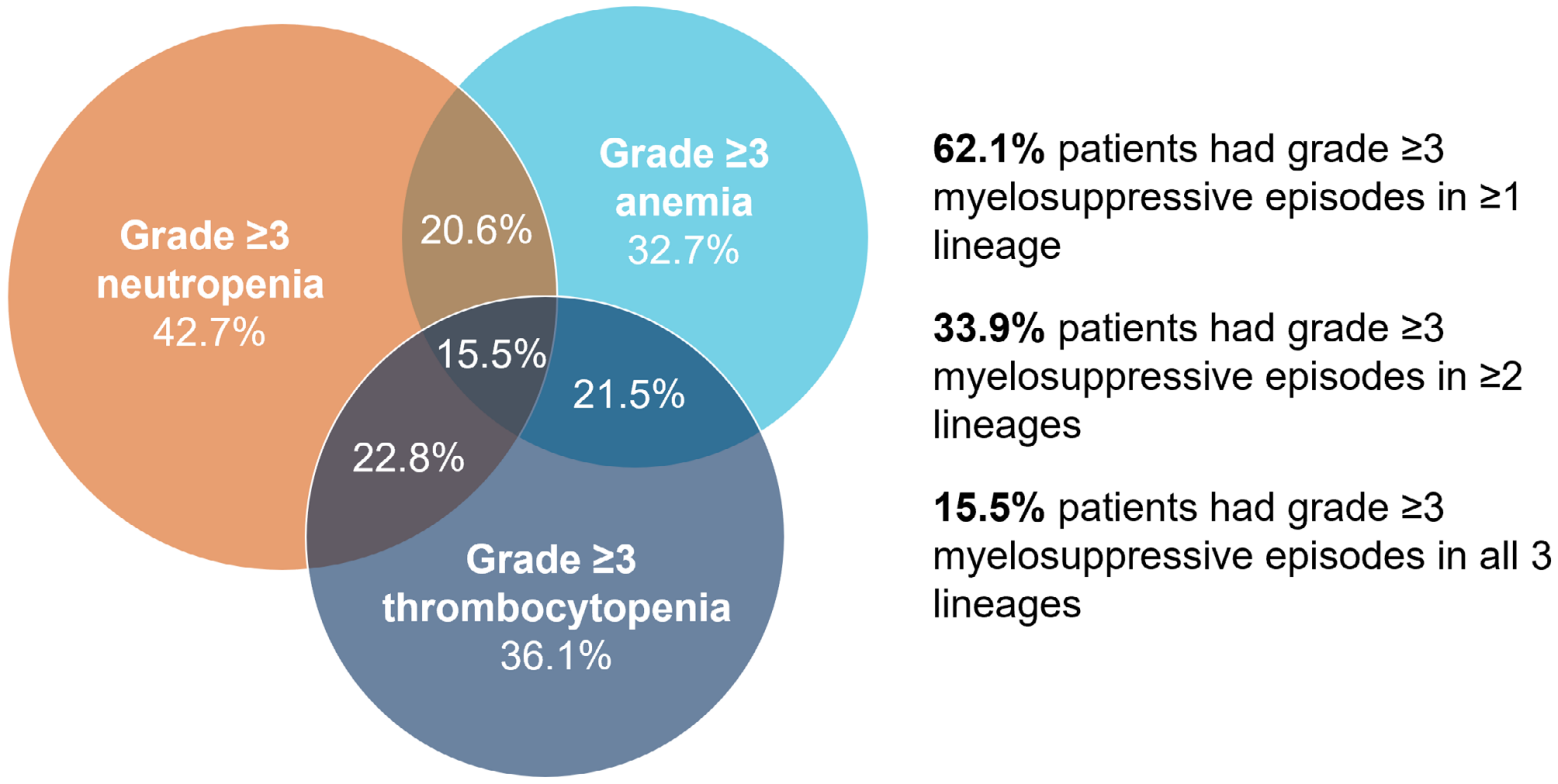
Grade ≥ 3 myelosuppressive episodes after chemotherapy. Percentages were calculated using the total number of patients with laboratory data available for neutropenia, thrombocytopenia, and anemia (*N*=1235) as the denominator.

Overall, 419 (33.9%) patients had grade ≥ 3 neutropenia, thrombocytopenia, and/or anemia episodes in at least two lineages (Figure 3). Among patients with laboratory data, 20.6% patients had grade 3 anemia and grade ≥ 3 neutropenia, 21.5% patients had grade 3 anemia and grade ≥ 3 thrombocytopenia, and 22.8% patients had grade ≥ 3 neutropenia and grade ≥ 3 thrombocytopenia. Nearly 1 in 6 patients (15.5%) patients had grade ≥ 3 myelosuppressive episodes in all three lineages.

**FIGURE 4:**
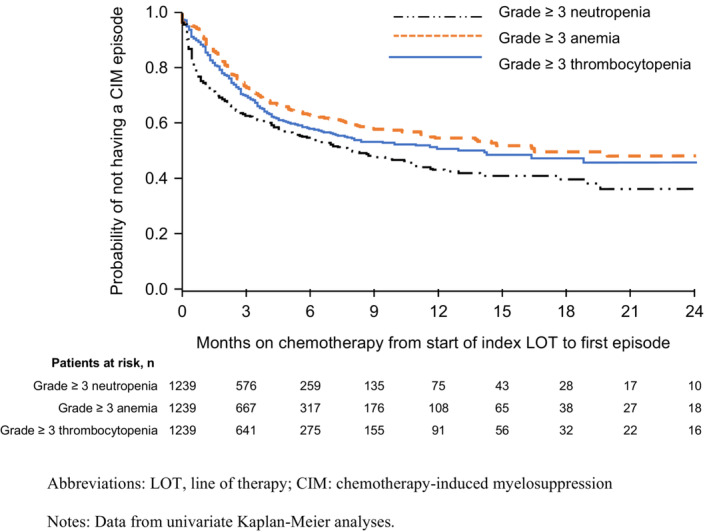
Time to first grade ≥ 3 myelosuppressive episodes from chemotherapy initiation. LOT, line of therapy; CIM, chemotherapy‐induced myelosuppression. Data from univariate Kaplan–Meier analyses.

At 3/6/12 months of cumulative time on chemotherapy, 37.2%/45.2%/57.0% of patients had grade ≥ 3 neutropenia, 26.7%/36.7%/45.3% had grade 3 anemia, and 29.5%/41.8%/49.0% had grade ≥ 3 thrombocytopenia (Figure 4).

### 3.3 Treatment patterns

The index treatment regimen was most often platinum/etoposide‐containing chemotherapy alone (64.3%) or platinum/etoposide‐containing chemotherapy in combination with immuno‐oncology treatment (22.4%; Table 2). During follow‐up, 94.6% of patients discontinued index treatment, and among those who discontinued, median time to discontinuation was 3.5 months. Approximately half (52.8%) of patients received at least one subsequent LOT. Among those who initiated subsequent therapy, median TTNT from the start of the index LOT was 4.6 months. The median number of regimens received was 2. Of 2336 regimens received across all LOTs, 52.2% were platinum‐based (alone or with immune‐oncology treatment) and 8.1% were topotecan‐containing chemotherapy (Figure S1).

**TABLE 2:** Treatment patterns.

	Patients with ES‐SCLC (*N* = 1239)
Treatment regimen in the index LOT, *n* (%)	
Platinum−/etoposide‐containing chemotherapy alone	797 (64.3)
Platinum−/etoposide‐containing chemotherapy + IO	277 (22.4)
Topotecan‐containing chemotherapy	29 (2.3)
Lurbinectedin	2 (0.2)
Other^a^	134 (10.8)
Discontinued index LOT^b^, *n* (%)	1172 (94.6)
Time to discontinuation of index LOT	
Among patients with event, months, mean (SD) [median]	4.4 (4.9) [3.5]
KM estimate^c^, median (95% CI)	3.6 (3.5, 3.8)
Initiated next therapy, *n* (%)	654 (52.8)
Time from start of index LOT to start of next LOT	
Among patients with event, months, mean (SD) [median]	6.2 (6.1) [4.6]
KM estimate^c^, median (95% CI)	6.9 (6.4, 7.5)
Number of LOTs during follow‐up, mean (SD) [median]	1.9 (1.1) [2]
Number of LOTs, *n* (%)	
1	585 (47.2)
2	381 (30.8)
3	158 (12.8)
4	81 (6.5)
≥5	34 (2.7)

Abbreviations: ES‐SCLC, extensive‐stage small cell lung cancer; KM, Kaplan–Meier; LOT, line of therapy; SD, standard deviation.

^a^ Includes other chemotherapy agents alone (including a platinum‐based agent without etoposide) or in combination with IO, chemotherapy plus other treatment, IO alone, and other treatment alone.

^b^ Includes 22 patients with a death date equal to the end of index LOT date.

^c^ KM figure available in Figure S2.

### 3.4 Supportive care use

Nearly all patients (94.0%) received some form of supportive care during follow‐up (G‐CSF = 89.7%, ESAs = 24.4%, IV volume expansion = 52.1%; Table 3). Almost one‐third (32.6%) of patients were eligible to receive RBC transfusion.

**TABLE 3:** Use of supportive care interventions across all LOTs.

	Patients with ES‐SCLC (*N* = 1239)
Eligible for transfusions, *n* (%)	
RBC (Hb <8 g/dL)	404 (32.6)
Platelet (platelets <10,000/μL)	46 (3.7)
RBC and/or platelet	418 (33.7)
Supportive care utilization, *n* (%)	
G‐CSF^a^	1112 (89.7)
ESAs^b^	302 (24.4)
IV volume expansion	646 (52.1)
G‐CSF, ESAs, and/or IV volume expansion	1165 (94.0)
G‐CSF administrations^c^, mean (SD) [median]	5.7 (6.8) [4]

Abbreviations: ESA, erythropoiesis‐stimulating agent; ES‐SCLC, extensive‐stage small cell lung cancer; G‐CSF, granulocyte colony‐stimulating factor; Hb, hemoglobin; IV, intravenous; LOT, line of therapy; RBC, red blood cell; SD, standard deviation.

^a^ Includes filgrastim (including biosimilars), TBO‐filgrastim, pegfilgrastim (including biosimilars), and sargramostim.

^b^ Includes epoetin alfa (and biosimilar).

^c^ Count of unique administration days for G‐CSF among all patients.

## 4 DISCUSSION

This retrospective EMR study evaluated the burden of myelosuppression and treatment patterns as well as associated supportive care use among chemotherapy‐treated patients diagnosed with ES‐SCLC in US community oncology practices. Results from this study suggest an unmet need for managing the burden of multilineage myelosuppression among patients receiving single‐lineage supportive care.

Clinical trials have reported a wide range of incidence rates for grade 3/4 neutropenia, anemia, and thrombocytopenia with first‐line platinum/etoposide regimens, with or without immunotherapy^32^, ^33^, ^34^, ^35^ and for second‐line topotecan.^36^, ^37^, ^38^ This study reported that chemotherapy‐induced myelosuppression was prevalent among patients in the real‐world community oncology setting; the most common any‐grade event being anemia, and the most common grade ≥ 3 event being neutropenia. Our findings are consistent with previous studies in the real‐world setting, which have reported that grade ≥ 3 myelosuppressive AEs are common among patients with ES‐SCLC treated with chemotherapy (56.6–64.1%)^39^, ^40^ and among patients with SCLC treated with chemotherapy (grade ≥ 3 = 60.9%; any grade with inpatient admission = 74.3%).^41^, ^42^ Rates of myelosuppressive events in this study were similar between the index LOT and all LOTs suggesting that myelosuppressive events occur early in the ES‐SCLC treatment course, rather than only during later LOTs. A notable proportion (33.9%) of patients experienced myelosuppressive episodes in at least two blood cell lineages, underscoring the multilineage burden of myelosuppression among patients with ES‐SCLC undergoing chemotherapy treatment.

Nearly all patients (94.6%) in this study discontinued their index LOT during follow‐up, with a median time to discontinuation of 3.5 months. This is largely in line with the recommended 4 cycles of initial therapy for ES‐SCLC based on National Comprehensive Cancer Network (NCCN) Guidelines® (although patients may receive up to 6 cycles depending on response and tolerability).^5^ Around half of patients went on to receive at least one further LOT, which is concordant with the 52.1% of elderly patients with SCLC who received a second or subsequent treatment in a recent Surveillance, Epidemiology and End Results (SEER)‐Medicare analysis, but higher than the 29.7% of SCLC patients with documented second‐ or later‐line treatment within the Providence St. Joseph Health EMR dataset.^41^, ^42^ Although NCCN Guidelines® now recommend platinum/etoposide plus immunotherapy as preferred options for patients with ES‐SCLC,^5^ the relatively low uptake of chemo‐immunotherapy in the current study can be attributed to the fact that more than 70% of patients had an index treatment date before 2019 (atezolizumab approved in March 2019 for the first‐line treatment of ES‐SCLC in combination with carboplatin and etoposide^32^; durvalumab approved in March 2020 for the first‐line treatment of ES‐SCLC in combination with either cisplatin/etoposide or carboplatin/etoposide^43^).

Supportive care use associated with myelosuppression was substantial in this study. Across all LOTs, nearly all patients received some form of supportive care, with close to 90% of patients receiving G‐CSF, and almost one‐quarter receiving ESAs. The rate was slightly higher than observed in previous real‐world studies for G‐CSF use (47%–84%) but within the reported range for ESA use (2%–27%).^39^, ^40^, ^41^, ^42^, ^44^ As reflected in the large variation among these studies, differences in methodology and reporting preclude any direct comparisons of healthcare resource use between studies.

Although frequently used, standard interventions for managing myelosuppressive AEs are suboptimal.^45^ As demonstrated in the high rates of cytopenia in more than one hematopoietic lineages, current supportive care measures that are lineage specific (G‐CSF for neutropenia, RBC transfusion or ESAs for anemia, platelet transfusion for thrombocytopenia) do not address the burden of myelosuppression in other lineages. Additionally, each of these interventions imparts their own set of risks, including musculoskeletal pain with G‐CSF, thromboembolic events with ESAs, and infections, immunological deregulation, and transfusion‐related reactions with use of blood products.^10^, ^46^, ^47^, ^48^ Also, these treatments do not proactively protect the bone marrow from chemotherapy‐induced damage.

Limitations of this study include those inherent with retrospective observational studies of EMR and/or administrative databases which were developed for non‐research purposes (e.g., practice management).^49^, ^50^ Because transfusion was not captured in the structured EMR data, eligibility to receive transfusion based on laboratory levels was assessed as a surrogate measure. This could potentially result in either over‐ or underestimation of transfusion rates given that decisions on whether to transfuse are based on other patient preference and clinical factors. The lack of inpatient data may also lead to underestimation of supportive care use and occurrence of myelosuppressive adverse events following hospital admission. On the other hand, the use of both ICD codes and laboratory values for identifying myelosuppression events rather than relying on physician‐reported adverse events likely increases the fidelity of the dataset compared with other sources such as medical charts or claims data. Additionally, the algorithm used to determine treatment regimens from the EMR data may not correctly identify LOT. Finally, the results of this study were based on data from community oncology settings and therefore may not be generalizable beyond this setting. Nonetheless, real‐world data are likely to be representative of clinical experience across a broad distribution of patients and can provide valuable insight into everyday treatment patterns and health outcomes.

## 5 CONCLUSION

Overall, the findings indicate that there is a high burden related to myelosuppression among chemotherapy‐treated patients with ES‐SCLC in the community oncology setting. Mitigating or preventing the myelotoxic effects of chemotherapy has the potential to reduce the burden of myelosuppression among patients with ES‐SCLC.

## AUTHOR CONTRIBUTIONS

**Lowell Hart:** Conceptualization (equal); data curation (equal); formal analysis (equal); funding acquisition (equal); investigation (equal); methodology (equal); project administration (equal); resources (equal); software (equal); supervision (equal); validation (equal); visualization (equal); writing – original draft (equal); writing – review and editing (equal). **Augustina Ogbonnaya:** Conceptualization (equal); data curation (equal); formal analysis (equal); funding acquisition (equal); investigation (equal); methodology (equal); project administration (equal); resources (equal); software (equal); supervision (equal); validation (equal); visualization (equal); writing – original draft (equal); writing – review and editing (equal). **Kristen Boykin:** Conceptualization (equal); data curation (equal); formal analysis (equal); funding acquisition (equal); investigation (equal); methodology (equal); project administration (equal); resources (equal); software (equal); supervision (equal); validation (equal); visualization (equal); writing – original draft (equal); writing – review and editing (equal). **Kathryn Deyoung:** Conceptualization (equal); data curation (equal); formal analysis (equal); funding acquisition (equal); investigation (equal); methodology (equal); project administration (equal); resources (equal); software (equal); supervision (equal); validation (equal); visualization (equal); writing – original draft (equal); writing – review and editing (equal). **Ray Bailey:** Conceptualization (equal); data curation (equal); formal analysis (equal); funding acquisition (equal); investigation (equal); methodology (equal); project administration (equal); resources (equal); software (equal); supervision (equal); validation (equal); visualization (equal); writing – original draft (equal); writing – review and editing (equal). **Trevor Heritage:** Conceptualization (equal); data curation (equal); formal analysis (equal); funding acquisition (equal); investigation (equal); methodology (equal); project administration (equal); resources (equal); software (equal); supervision (equal); validation (equal); visualization (equal); writing – original draft (equal); writing – review and editing (equal). **Lorena Lopez‐Gonzalez:** Conceptualization (equal); data curation (equal); formal analysis (equal); funding acquisition (equal); investigation (equal); methodology (equal); project administration (equal); resources (equal); software (equal); supervision (equal); validation (equal); visualization (equal); writing – original draft (equal); writing – review and editing (equal). **Huan Huang:** Conceptualization (equal); data curation (equal); formal analysis (equal); funding acquisition (equal); investigation (equal); methodology (equal); project administration (equal); resources (equal); software (equal); supervision (equal); validation (equal); visualization (equal); writing – original draft (equal); writing – review and editing (equal). **Lucio Gordan:** Conceptualization (equal); data curation (equal); formal analysis (equal); funding acquisition (equal); investigation (equal); methodology (equal); project administration (equal); resources (equal); software (equal); supervision (equal); validation (equal); visualization (equal); writing – original draft (equal); writing – review and editing (equal).

## FUNDING INFORMATION

This study was funded by G1 Therapeutics, Inc.

## CONFLICT OF INTEREST STATEMENT

L. Lopez‐Gonzalez (at the time of this study) and H. Huang are employees of G1 Therapeutics, Inc. L. Hart, K. Boykin, R. Bailey, T. Heritage, and L. Gordan are employees of the Florida Cancer Specialists & Research Institute, which received funding from G1 Therapeutics, Inc., for this work. A. Ogbonnaya and K. Deyoung are employees of Xcenda, which received funding from G1 Therapeutics, Inc., for this work.

## PREVIOUS PRESENTATIONS

Part of this work was presented as a poster at the AMCP Annual Meeting 2022 (Chicago, IL, USA; March 29–April 1, 2022).

## THIRD PARTY SUBMISSIONS

Barbara Blaylock from Blaylock Health Economics LLC provided editorial and publication support funded by G1 Therapeutics, Inc. All authors have approved the manuscript and authorized Barbara Blaylock to submit the manuscript on their behalf.

## Supporting information




Figure S1
Figure S2

## Data Availability

This study used data from the Florida Cancer Specialists and Research Institute electronic health records, which are not publicly available.
