# Supplementary material for: Burden of chemotherapy‐induced myelosuppression among patients with extensive‐stage small cell lung cancer: A retrospective study from community oncology practices
Source: Cancer Med. 2023 Mar 31;12(8):10020–30. doi: 10.1002/cam4.5738 (PMC10166910; doi:10.1002/cam4.5738)
Supplement: Supplementary file 1 — Figure S1 Figure S2 [file CAM4-12-10020-s001.docx]

**Supplemental materials**

**Figure S1. Treatment regimens across all LOTs^†^**

^
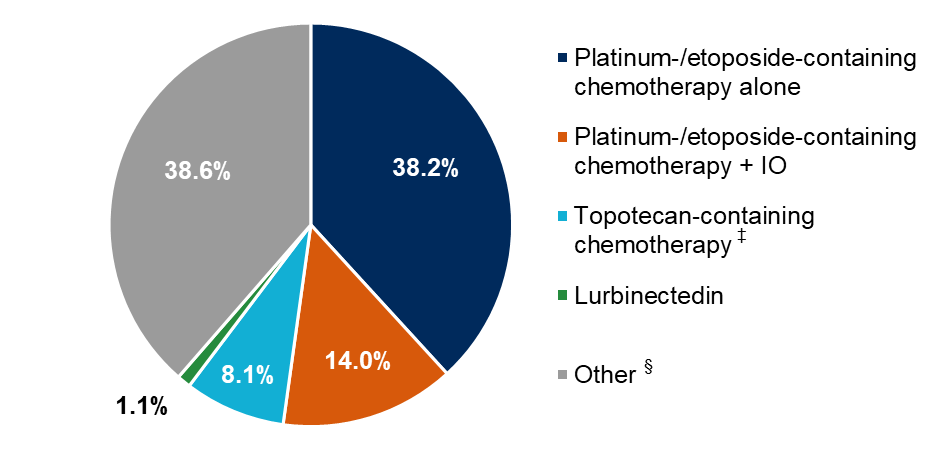
^

Abbreviations: IO, immuno-oncology treatment; LOT, line of therapy.

Notes: ^†^ Unit of assessment is count of regimens (N=2,336). ^‡^ Includes two patients who received topotecan plus IO (atezolizumab). ^§^ Includes other chemotherapy agents alone (including a platinum-based agent without etoposide) or in combination with IO, chemotherapy plus other treatment, IO alone, and other treatment alone.

**Figure S2. Kaplan-Meier plots of (A) time to discontinuation of the index therapy and (B) time to next therapy**

**(A)**


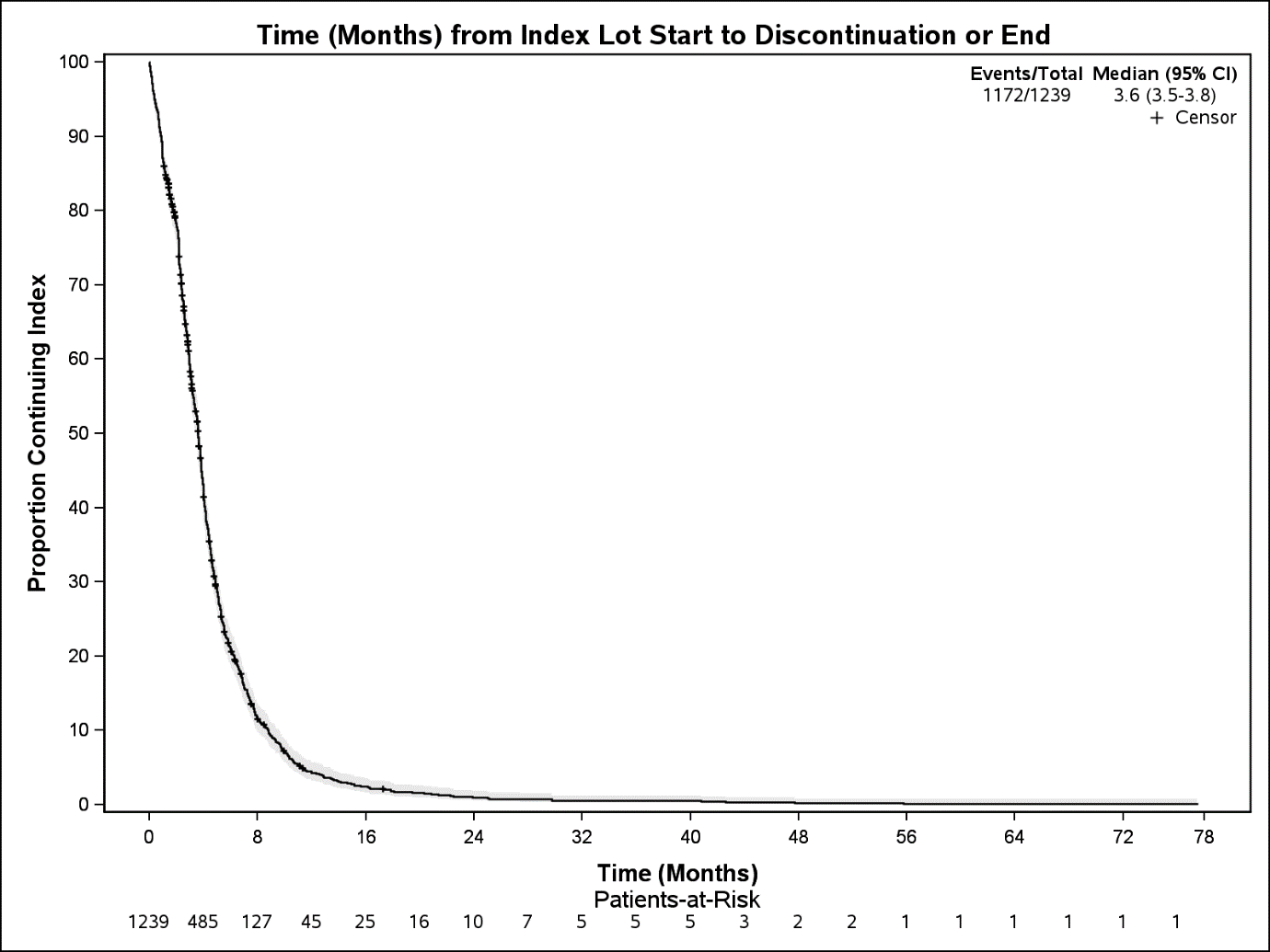


**(B)**


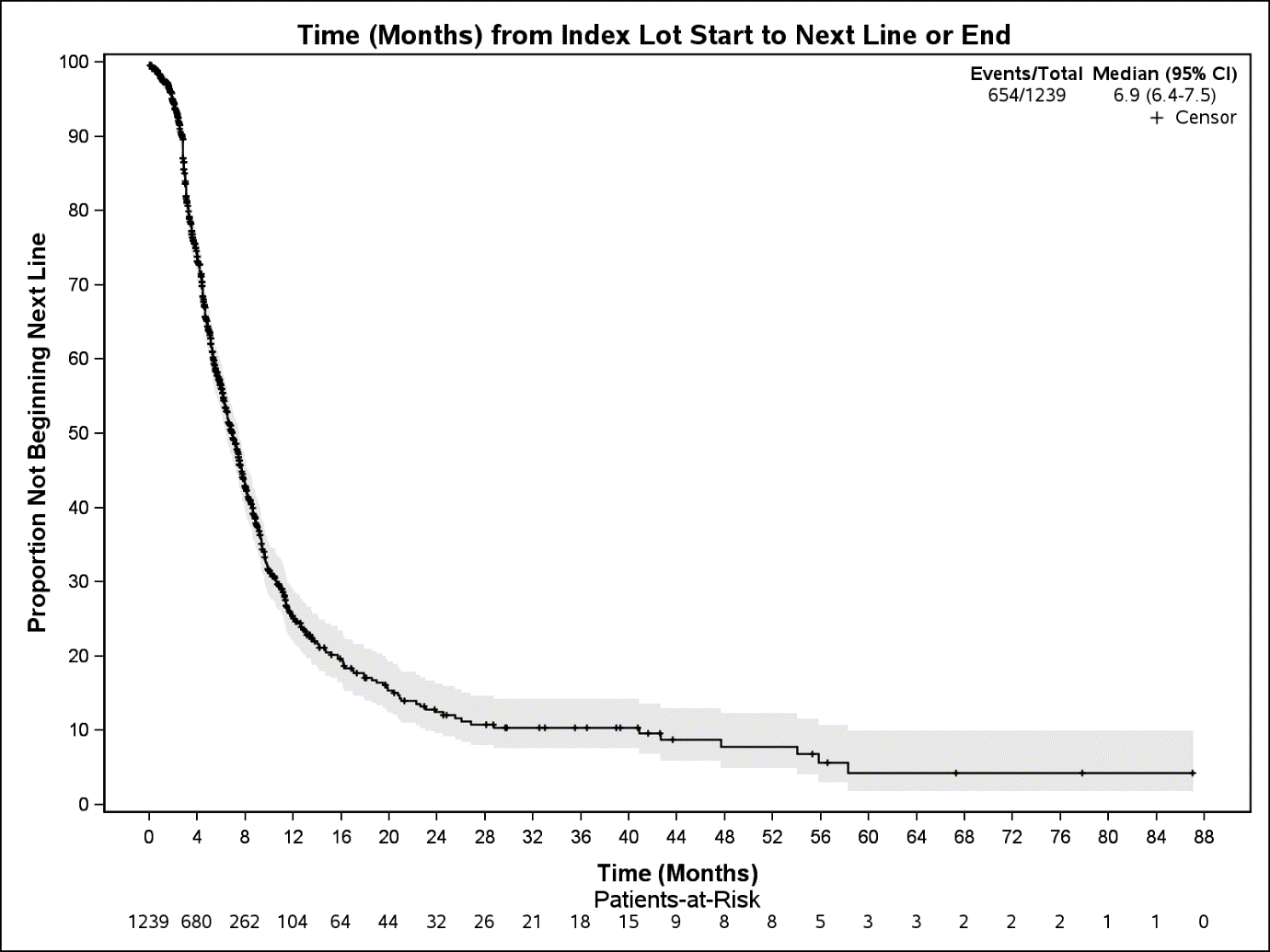


Abbreviations: LOT, line of therapy.

Notes: Data from univariate Kaplan-Meier analyses.
